# Tuberculosis in the Female Genital Tract

**DOI:** 10.7759/cureus.28708

**Published:** 2022-09-02

**Authors:** Himanshi Kesharwani, Shazia Mohammad, Pranav Pathak

**Affiliations:** 1 Obstetrics and Gynaecology, Jawaharlal Nehru Medical College, Datta Meghe Institute of Medical Sciences, Wardha, IND

**Keywords:** genital tuberculosis, gutb, fgtb, pregnancy, mdr-tb, infertility, fallopian tube, menstrual dysfunction

## Abstract

Genitourinary tuberculosis (GUTB) is caused by *Mycobacteria tuberculosis *bacilli and is typically secondary to tuberculosis (TB) of the lungs. The spread largely occurs through the haematogenous route.* Mycobacterium tuberculosis* complex infections frequently cause the symptoms by reactivation of previously dormant tuberculous bacilli. Particularly in underdeveloped nations, female genital TB (FGTB) continues to be a key contributor to tubal blockage and infertility. It damages genital organs, which results in abnormal menstruation and infertility. FGTB is a chronic condition that manifests as mild symptoms. Almost all cases of genital TB include the fallopian tubes, which, together with endometrial involvement, render patients infertile. There may be asymptomatic cases. In order to save women from invasive surgery, it is vital to keep in mind the extremely rare but critical role of FGTB in the differential diagnosis of any malignancy. A thorough physical examination, careful history collection, and careful use of tests are done to arrive at a diagnosis. Hysterosalpingography has been recognised as the most accurate method for detecting FGTB and as the gold standard screening test for determining tubal infertility. Recently, there have been numerous improvements and modifications to FGTB management. The primary treatment for TB is a multidrug anti-TB regimen, while surgery may be necessary in more severe cases. Even after receiving multimodal therapy for TB, infertile women with genital TB have low conception rates and a significant risk of complications like ectopic pregnancy and loss.

## Introduction and background

**Figure 1:**
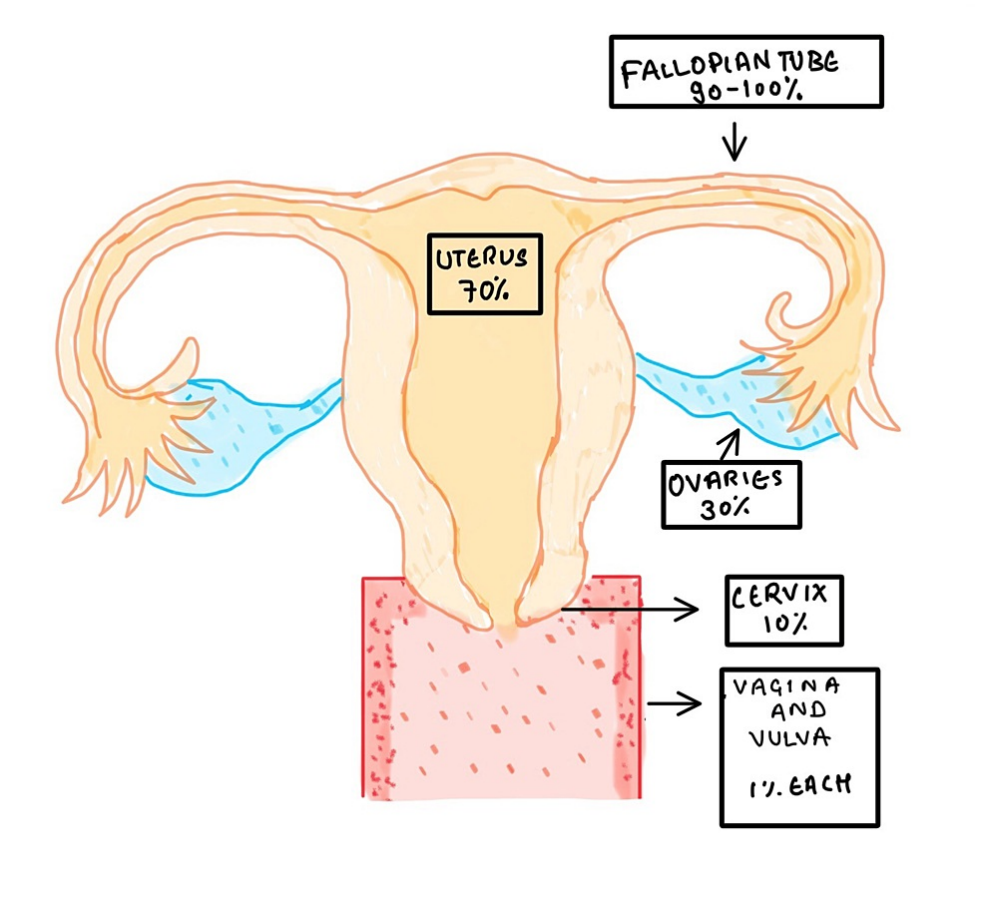
Percentage-wise distribution on the basis of the area affected

Tuberculosis (TB) is considered one of the significant causes of mortality by an infectious agent. Approximately 2.5 million new infections were reported in India in the year 2021 [1]. As per the Global Tuberculosis Report, 25% of the world's population suffers from 'latent TB', meaning they have been exposed to *Mycobacterium tuberculosis* although they did not get infected from it and are not capable of its further transmission. TB is a crucial problem in the health domain worldwide; despite an effective diagnosis and treatment, a decline in the mortality trend has not been completely achieved yet [2]. The estimated percentage of lifetime risk of falling ill for individuals having strong immunity is about 5-15% [3]. One kind of extrapulmonary presentation of TB is female genital TB (FGTB), which accounts for 5% of all female pelvic infections and 10% of pulmonary TB cases [4,5]. It is more commonly seen in developing countries, causing manifestations like chronic pelvic inflammatory disease (PID) and infertility [6]. FGTB can result in irreversible harm to the genital tract of women at progressive levels. Therefore, for the sake of prevention, early detection and management at the subclinical level is the only way to stop enduring harm and sequelae of the genital area of the woman with a thriving result of pregnancy [7]. Patients who already suffer from HIV infection or any other form of acquired or innate immuno-compromised states are more at risk. Malnutrition, poor access to healthcare, and poverty are additional risk factors. The uterine tubes are most frequently damaged (90-100%), followed by the uterus (70%), ovaries (30%), cervix (10%), and rarely the vulva and vagina (1% each) as shown in Figure 1 [8]. FGTB has been identified as an essential causative factor for infertility in places with a significant incidence of TB [9].

## Review

Etiopathogenesis

Infection of the genital area is due to secondary infection from pulmonary TB. It spreads directly from the hematogenous or lymphatic route and is typically seen in immuno-compromised individuals. It is also seen in people who have been suffering from long-lasting diseases like HIV and also coronavirus disease 2019 (COVID-19) [5]. Genital TB can be directly introduced during sexual contact with a male partner who has genitourinary TB (GUTB), which is a rare primary infection. There have been reports of infection spreading rapidly from the vagina, cervix, and vulva [10]. Ovarian cancer can develop due to peritoneal adhesions, nodules, and ascites, which are seen in abdominopelvic TB [11]. Likewise, cervical TB may mimic the cervical malignancy presented by symptoms like discharge from the vagina that is not normal [12]. GUTB is usually observed after the development of primary pulmonary TB or extrapulmonary lesion, for example, kidneys, meningeal area, skeletal system, and GI system through four routes: blood (in this, the pulmonary area will be the primary focus), direct spread, outspread through lymph nodes, and in rare cases, the reproductive tract through sexual transmission [13].

Clinical presentation

Most cases of genital TB have been seen in fertile people between the ages of 20 and 45 years [14]. Although genital TB may show a variety of clinical signs, the condition is typically asymptomatic and is initially detected during investigations for infertility [15,16]. The most common symptom seen is infertility; additional symptoms are irregular changes in the menstrual cycle (such as hypomenorrhea, heavy menstrual bleeding, dysmenorrhoea, and metrorrhagia), pain in the pelvis area, and discharge from the vagina that is not normal. Sometimes it can be asymptomatic also. TB of the reproductive tract presents with complaints that resemble cancer of the endometrium, namely post-menopausal bleeding, continuous leucorrhoea, and pyometra in postmenopausal women as mentioned in Table 1 [5,13].

**Table 1:** Female genital tuberculosis common signs and symptoms TB: tuberculosis; PTB: pulmonary tuberculosis Table Source: Sharma et al. [5] (Open Source)

Symptoms	Signs	General systemic symptoms	Abdominal examination	Menstrual symptoms	Vaginal examination	Unusual findings
No symptoms (10%)	No sign (10%), lymphadenopathy (in lymph nodes TB), crackles on chest auscultation (PTB), raised temperature	Pyrexia, anorexia, weight loss, feeling unwell, malaise	Vague or definite abdominal or pelvic lump, ascites, doughy feel of the abdomen	Puberty menorrhagia, heavy menstrual bleeding (in early stage), postmenopausal bleeding, oligomenorrhoea, hypomenorrhea, amenorrhoea (primary and secondary), dysmenorrhoea	Soft, tender enlarged uterus (pyometra), tenderness and induration in the fornices, lump in adnexa, fullness, and tenderness in the pouch of Douglas	Solid lesions on external genitalia, ulcers on external genitalia, genital fistula Infertility (primary and secondary), abdominal or pelvic mass, abdominal and pelvic pain, acute abdomen, vaginal discharge, Douglas ulcers or growth, urinary incontinence, or faecal incontinence

Causes of infertility

Bilateral fallopian tubes are affected, resulting in infection and inflammation of the fallopian tubes along with blockage of the tubes, in almost all cases of FGTB [9]. Uterine endometrium is affected by TB due to continuous endometrial destruction and development of synechiae inside the uterus (Asherman’s syndrome) further leading to the hostility of endometrium through rising levels of tumor necrosis factor (TNF)-alpha, IL-2 measures causing recurrent implantation failure and recurrent miscarriages known as latent FGTB [17]. Ovaries can also be destroyed by TB, along with oophoritis with poor reserve of the ovary and expanded requirement of gonadotropins for starting the process of ovulation [18].

Differential diagnosis

It is very important to also consider the other diseases that show similarities in presentation with FGTB like an inflammatory disease of the pelvis, ectopic conception, and cysts in the ovaries. Other examples include malignancy of ovaries, ovarian and cervical TB, and elephantiasis of the vulva in vulva TB as given in Table 2 [6,19].

**Table 2:** On the basis of the site involved, the differential diagnosis of the FGTB differs FGTB: female gential tuberculosis Table Source: Grace et al. [6] (Open Source)

Site of involvement	Differential diagnosis
Tuberculous salpingitis	Pelvic inflammatory disease, Ectopic pregnancy, Ovarian cyst, Endometriosis, Carcinoma of the colon, Diverticulitis
Endometrial tuberculosis	Dysfunctional uterine bleeding, Endometrial carcinoma
Ovarian tuberculosis	Ovarian malignancy
Cervical tuberculosis	Carcinoma of the cervix
Vulval tuberculosis	Elephantiasis vulva

Investigations

The highest probability of having FGTB is in individuals who have a long-lasting pelvic inflammatory disease and are not responding to antibiotic treatment, idiopathic infertility, irregular cycles of menses or bleeding after menopause, and constant discharge from the vagina (wherein genital malignancy has been eliminated). Predisposing factors include interaction with a pulmonary TB patient with a positive smear test, prior TB infection, past history of TB infestation, fresh travel to the endemic region, poor socio-economic status, and residing in a population that has HIV and drug abusers. There has to be a higher level of clinical suspicion, a thorough medical history, and a thorough physical examination to verify FGTB infection. A number of tests are used to confirm TB infection and imaging techniques for typical structural abnormalities are crucial for the diagnosis [5]. WHO defines that extrapulmonary TB diagnosis is confirmed by one specimen with a positive culture, a positive histology result, or significant clinical evidence of active extrapulmonary TB [2].

Baseline Investigations

Baseline testing should include a general examination to rule out the presence of TB somewhere else in the body, a chest X-ray, a tuberculin skin test (TST), an erythrocyte sedimentation rate (ESR), and a full blood count. A tuberculin skin test is only marginally useful in places with increased TB prevalence and where routine Bacillus Calmette-Guérin (BCG) vaccine immunization is practised. A tuberculin skin test can also result as positive by mistake (non-TB mycobacterial infection) and negative by mistake (patients having steroid medication, existing together with HIV infection, new TB infection, chronic failure of kidney, and those who have typhoid fever, typhus, brucellosis, leprosy, or pertussis) [5].

Hysterosalpingography (HSG)

Genital TB shows an association with specific changes in the structure of the affected organ; HSG is a valuable tool for observing deformity. On HSG, tubal TB manifestations range from nonspecific changes such as dilation of the tube, obstruction of the tube, irregularities in contours, diverticulum outflow (nodular salpingitis), hydrosalpinx to specific patterns such as "tampon" and "tubular". Golf club tube, cobblestone tube, bead tube, leopard tube, occlusion in the tube, as well as synechiae formation in the peritubular area, can manifest as a straight spill corkscrew appearance and halos around the tube [20]. TB should be highly suspected if there is the presence of adhesion and obstruction of the fallopian tubes seen in the zone of transition, which is in the middle of the isthmus and ampulla or more than one strictures, lymph node calcification, not regular, linear, or single calcification at the adnexal area [21,22]. Tuberculous uterine changes could be observed as nonspecific characteristics like adhesion development, pus-filled uterus, venous and lymphatic intravasation or particular features like "collar segment," "T-abnormal," and "pseudocornuate" uterus [23]. Because the stratified epithelium of the ectocervix inherently resists bacterial invasion, cervical TB is uncommon and typically related to endometrial and fallopian tube TB. If the cervix is involved, it appears on an HSG as a feathery appearance, diverticular outpouching, uneven shapes, cervical distortion, and a serrated endocervical canal [24].

Ultrasonogram

The fallopian tubes may dilate, thicken, and fill with clear fluid or a dense substance called a hydrosalpinx. The endometrium may have intrauterine adhesions, calcification or fibrosis, heterogeneous appearance, and hyperechoic areas representing malformed uterine cavities [22]. Results may differ from a normal examination to anomalies like an endometrium that is thin or thick, changes in blood vessels in the endometrium between stimulated menstrual cycles, endometrial calcification, changes in uterine arteries, the release of tubal fluid and peritoneal, during mid-cycle flux and unstable ovaries homogeneous growth and adnexal fixation. Some of the more specific findings are the presence of sacs with hydrosalpinx, endometrial fluid, and echogenic rims [25].

Laparoscopy

In spite of the fact that laparoscopy has a high tendency to invade deeply into the tissue, it helps to visualise the ovaries, fallopian tubes, peritoneal cavity, and biopsy of tuberculous lesions. The main benefit of merging hysteroscopy and laparoscopy is not limited to excluding the involvement of endometrial, even so for the purpose of performing various interventions; for example, breaking of adhesion or stimulation of the endometrium with estrogen [5]. Laparoscopic findings show genital TB that may be different from natural appearances up to superficial TB, fimbrial phimosis, fimbrial block, hyperplasia, tubal beads, peritubular adhesions, periovarian adhesions, tubovarian masses, hydrosalpinx, and rigid tube [26].

Histopathological Examination

Histopathological examination shows particular features of TB, such as granulomatous caseous lesions, as a sign of infection. The presentation of characteristic caseous granulomas with giant epithelial cells suggests TB. But similar lesions can also be seen in syphilis, leprosy, rheumatoid arthritis, systemic lupus, erythematosus, pneumonia, and, in rare cases, sarcoidosis [26]. Tubal tissue sections of the fallopian tube show caseation and acid-fast bacilli. In TB of ovaries, caseation is unusual, but granulomas can be found commonly visible in the cortical region of the ovary [27]. Granulomas of epithelioid type may be found in cervical TB along with caseation, whereas acid-fast bacilli are an unusual condition in vaginal as well as vulvar TB. Because uterine TB is often misdiagnosed as cancer, it is important to distinguish between the two at an early stage [28].

Bacteriological Evaluation

Isolation of TB bacilli is the definitive diagnosis of TB isolation that can be made using techniques like microscopy and culture. Microscopy for acid-fast bacilli is a rapid test for diagnosis but with variable sensitivity [29]. For diagnostic testing, on the first day of menstruation, blood and fluid can be removed from the genital tract [30]. A positive result from the acid-fast stain for endometrial curettage needs 10 organisms per millilitre [31]. The presence of *Mycobacterium tuberculosis* in culture supports the diagnosis of TB. Middlebrook 7H10 medium or Lowenstein-Jensen (LJ) medium, both of which are egg-based, are frequently used for solid cultures, and liquid cultures are based on modified Brookenbrook using BACTEC Mycobacterium 960 Automatic Growth Indicator Tubes (MGIT 960) (Becton, Dickinson and Company, Franklin Lakes, New Jersey, United States) and oxygen sensitive fluorescence detection technology [32].

The evaluation and application of molecular methods for TB detection have recently increased. Results from the nucleic acid amplification test (NAAT) are available soon. A quick molecular technique called polymerase chain reaction (PCR) can quickly find *Mycobacterium*-specific nucleic acid sequences in tissue samples from FGTB patients. PCR assays can detect dead bacilli with <10 bacilli per millilitre and have a test time of 8-12 hours [33]. The WHO prohibits the use of serological tests in persons who are ill from active TB, regardless of the status of HIV [34].

Management

TB Notification

According to the India TB Notification Policy, the tuberculosis notification portal, Nikshay, must be used to notify all TB patients, including those with FGTB . Patients with TB, which also includes patients with FGTB, may also take advantage of the government's Nikshay Poshan Yojana, which provides these patients with an amount of rupees 500 per month to purchase rations while their management is still ongoing [9].

Medical Treatment

Similar to pulmonary TB, FGTB treatment lasts for a total of six months. During the first two months of the intensive phase, four medications rifampicin (R), isoniazid (H), pyrazinamide (Z), and ethambutol (E) are administered per oral. In the continuation phase, a three-drug regimen (instead of the prior two) was administered daily for the next four months: rifampicin (R), isoniazid (I), and ethambutol (E). For individuals with primary or secondary FGTB that is resistant to drugs, an extended oral regimen is prescribed for 18-20 months or a short regimen for at least 9-12 months. Further details about the management of GUTB are given in Table 3. Every woman should be closely watched for medication compliance and any adverse effects, including hepatotoxicity, eye toxicity (ethambutol), and peripheral neuropathy (isoniazid). Any neuritis can be treated with pyridoxine, and if hepatotoxicity is anticipated, liver function tests should be carried out [35].

**Table 3:** Management of female genital TB FGTB: female genital TB; ATT: anti-tubercular therapy Table Source: Guidelines on Extrapulmonary tuberculosis for India, Ministry of Health and Family Welfare, Government of India, and WHO [36].

	First-line treatment for adults and children with genitourinary TB
Drugs	2HRZE/4HRE
Duration	Six months
Referral	A gynecologist's evaluation is necessary to make the diagnosis and address any problems. Women who present with infertility alone should only begin empirical ATT after receiving a specialist's evaluation.
Follow-up	Assess response to treatment at completion of ATT
Surgery	Surgery is not typically required for FGBT primary care, but it may be for large, persistent tubo-ovarian abscesses. Due to the numerous adhesions and risk of infection recurrence, surgery in FGBT is linked to greater complication rates. Following ATT, it is occasionally possible to surgically reconstruct the tubal anatomy in infertile women. However, a long-term effect of FGBT may be infertility, which may be irreversible. It is not required to administer another round of ATT to women who are still unable to conceive after receiving treatment for FGBT.

Surgical Treatment

Surgery is discouraged in FGTB due to numerous risks, and anti-tubercular therapy (ATT) is recommended. Only limited surgery can be done in the form of drainage of the abscess. However, diagnostic laparoscopy and hysteroscopy are performed initially for diagnosis and later to predict the prognosis. Then, when TB therapy is over, additional infertility treatment can be done.

The following is a strategy for infertility treatment: Firstly, normal conception is attempted if the tubes are open during laparoscopy and the endometrium is normal during hysteroscopy, or ovulation induction medication is used. Otherwise, in vitro fertilization and embryo transfer (IVF ET) is conducted with better outcomes and a pregnancy rate of up to 25% if there is a blockage in the fallopian tubes and the endometrium is receptive (typically) [9].

TB and Pregnancy

In pregnancy, pulmonary as well as extrapulmonary TB may manifest. Even in the first trimester of pregnancy, drug-susceptible TB should be managed with four medications; rifampicin (H), isoniazid (R), pyrazinamide (Z), and ethambutol (E) for two months and three drugs rifampicin (H), isoniazid (R) and ethambutol (E) for four months. Patients with drug-resistant TB should avoid getting pregnant while on reserve medications. Due to the potential teratogenicity of reserve medicines, medical termination of pregnancy (MTP) is recommended for multi-drug resistant TB (MDR TB) patients during the first trimester [9].

Prevention

Strategies to lower the risk of mycobacterial exposure are part of primary TB prevention. Therefore, it's necessary to inform patients with pulmonary TB about the need to maintain good hygiene of the respiratory system at home and in public places, as well as the continuation of the recommended therapy. Adopting safe sex habits can lower the risk of genital infection, specifically for genital TB. The BCG vaccine is used as a preventative measure in nations with a high TB burden, such as India. Although the BCG vaccination can protect against the onset of advanced TB up to 80% of the time, its preventive efficacy differs significantly amongst demographic groups [37].

## Conclusions

Latent FGTB causes recurrent pregnancy loss (RPL), and infertility, which is the major cause of concern in developing countries like India. However, IVF ET is a good option for tubal blockage with normal endometrium with a promising result. Surrogacy and adoption can be recommended if the endometrium and ovaries are damaged. Treatment with anti-tubercular drugs for 12 months has shown results of spontaneous conception. Genital TB requires a high index of suspicion and proper evaluation for better management. Women who are fertile and in the age group of 20-45 years of age are advised to have safer sex practices. ATT initiated in the early stage of the disease would help to have a better prognosis; those women who wish to have children should consider the option of in-vitro fertilisation with embryo transplant.
